# How to use CRISPR/Cas9 in plants: from target site selection to DNA repair

**DOI:** 10.1093/jxb/erae147

**Published:** 2024-04-22

**Authors:** Adéla Přibylová, Lukáš Fischer

**Affiliations:** Department of Experimental Plant Biology, Faculty of Science, Charles University, Viničná 5, 12800, Prague 2, Czech Republic; Department of Experimental Plant Biology, Faculty of Science, Charles University, Viničná 5, 12800, Prague 2, Czech Republic; University of South Bohemia in České Budějovice, Czech Republic

**Keywords:** Cell cycle, cleavage, CRISPR/Cas, DNA repair, editing, mutagenesis, plants, post-cleavage trimming, staggered ends

## Abstract

A tool for precise, target-specific, efficient, and affordable genome editing is a dream for many researchers, from those who conduct basic research to those who use it for applied research. Since 2012, we have tool that almost fulfils such requirements; it is based on clustered regularly interspaced short palindromic repeats (CRISPR)/CRISPR-associated protein (Cas) systems. However, even CRISPR/Cas has limitations and obstacles that might surprise its users. In this review, we focus on the most frequently used variant, CRISPR/Cas9 from *Streptococcus pyogenes*, and highlight key factors affecting its mutagenesis outcomes: (i) factors affecting the CRISPR/Cas9 activity, such as the effect of the target sequence, chromatin state, or Cas9 variant, and how long it remains in place after cleavage; and (ii) factors affecting the follow-up DNA repair mechanisms including mostly the cell type and cell cycle phase, but also, for example, the type of DNA ends produced by Cas9 cleavage (blunt/staggered). Moreover, we note some differences between using CRISPR/Cas9 in plants, yeasts, and animals, as knowledge from individual kingdoms is not fully transferable. Awareness of these factors can increase the likelihood of achieving the expected results of plant genome editing, for which we provide detailed guidelines.

## Teaser

This review highlights often neglected factors important for successful CRISPR/Cas9 genome editing, such as varying *Sp* Cas9 cleavage activities and cell cycle stage, crucial in determining DNA repair and mutagenesis outcomes.

## Introduction

Throughout the text, we have divided the cited literature into two categories: plants and others, indicated by the superscript P and O, respectively, after the citation.

In the last few decades, invaluable tools have been developed for plant breeding and functional analysis of plant genes. The first milestones were zinc-finger nucleases (ZFNs; Kim *et al.*, 1996^O^) and later on transcription activator-like effector nucleases (TALENs; Boch *et al.*, 2009^P^). In both cases, designing the editing tool for a specific target sequence is time- and cost-consuming since these tools are based on programmable DNA-binding protein domains, so specific gene segments have to be designed and synthesized for each target (Beying *et al.*, 2021^P^).

In recent years, the clustered regularly interspaced short palindromic repeats (CRISPR)/CRISPR-associated protein (Cas) systems have emerged as a revolutionary powerful genome editing tool that outperforms previous tools. The technology is based on the adaptive immune system of bacteria, which makes it possible to target specific DNA sequences in the genomes of different organisms (Garneau *et al.*, 2010^O^; Gasiunas *et al.*, 2012^O^; Jinek *et al.*, 2012^O^). The main advantage of CRISPR/Cas technology lies in its simplicity and cheapness because the sequence specificity is determined by a 20 nt long region of a programmable RNA component (guide RNA), whose sequence can be simply adopted by PCR using primers specific for the selected locus (Gasiunas *et al.*, 2012^O^; Jinek *et al.*, 2012^O^).

In the basic set-up, genome editing by all the above-mentioned tools (ZFN, TALEN, and CRISPR/Cas) is based on the targeted induction of double-strand break (DSBs) by endonucleolytic activity of the editors. In the case where the subsequent DNA repair is error free, the target site can be cleaved again (and again) until a mutation occurs that prevents recognition of the repetitive site; that is, editing occurs. In this most frequently used approach, the likeliness of and type of edits depend on the target sequence, its surroundings, and chromatin state, but also on the state of the edited cell since different DNA repair pathways are available depending on the cell type and cell cycle phase at the moment of DSB generation.

This review explores the complexity of the *Streptococcus pyogenes*-derived system (*Sp*Cas9) and offers a comprehensive exploration of its mechanisms, from the recognition of the PAM (protospacer adjacent motif) sequence to the release of *Sp*Cas9 from its target site and the mechanisms that repair the DSB created by *Sp*Cas9. In the following five sections of our review, we first introduce the fundamentals of CRISPR/*Sp*Cas9 (see ‘Fundamentals of CRISPR/Cas9’ and describe pathways that may be involved in repairing DSBs (see ‘Fundamentals of DNA repair’). In ‘Factors affecting the overall efficacy and outcome of the CRISPR/*Sp*Cas9 mutagenesis’, we focused on various factors affecting the efficiency and outcome of the CRISPR/*Sp*Cas9 mutagenesis. ‘Guide for using CRISPR for editing’ builds on the knowledge from the former sections and describes practical hints on how to use CRISPR/*Sp*Cas9 for editing in plants. Finally, in ‘Other uses of the *Sp*Cas9 system’, we briefly describe other possible system applications.

Overall, we provide a roadmap for researchers navigating the expanding field of CRISPR technologies. In essence, this review seeks to be a valuable resource for both novice and experienced researchers in the field, offering nuanced insight into the multifaceted world of genome editing with CRISPR/Cas9.  To date, this is the first plant-oriented review that links CRISPR/*Sp*Cas9 to DNA repair, emphasizing the role of the cell cycle. Understanding the basic processes that govern the functionality of CRISPR/Cas is essential for exploiting its potential in a variety of applications.

## Fundamentals of CRISPR/Cas9

**Fig. 1.**
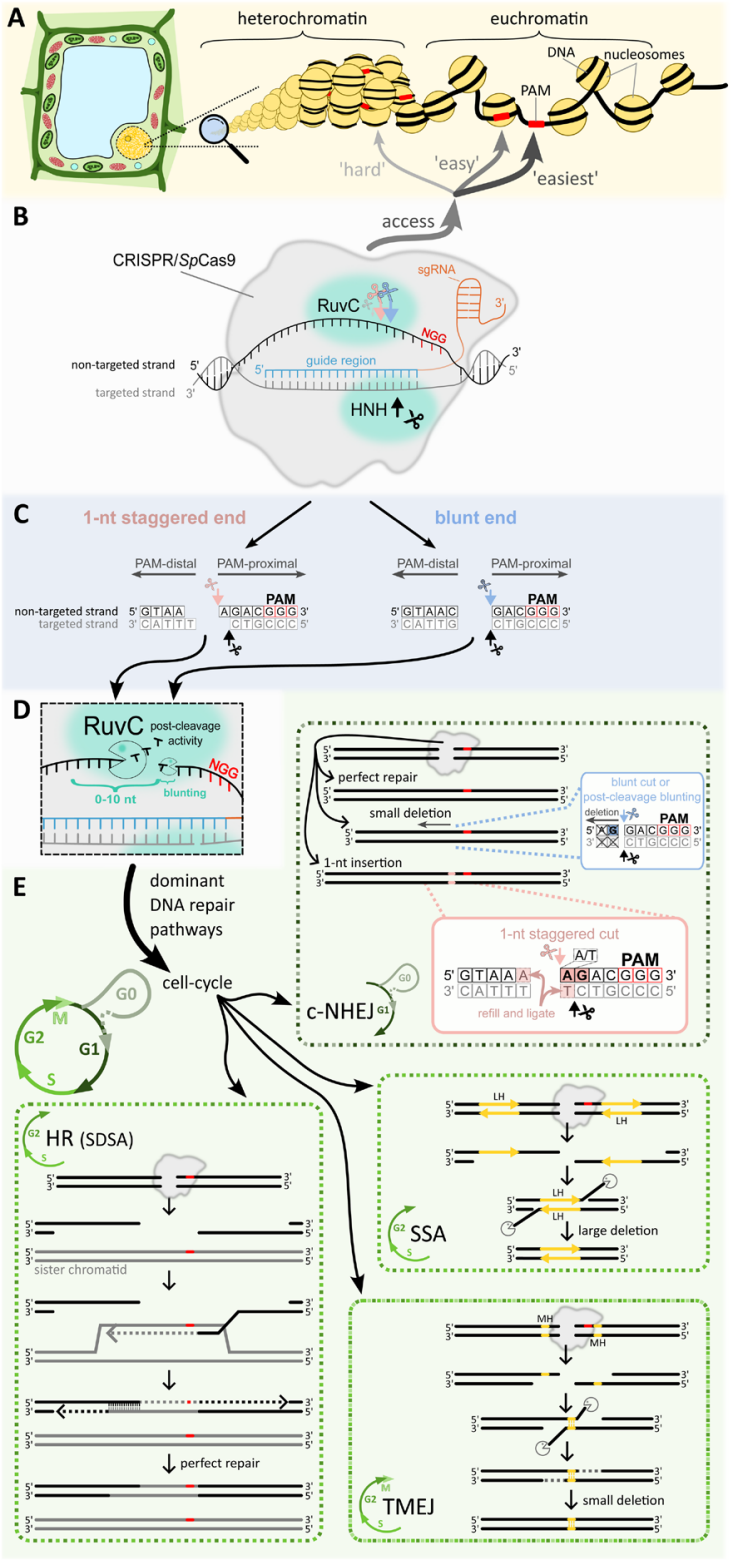
Overview of CRISPR/*Sp*Cas9 mutagenesis in a plant cell. (A) The illustration shows two main chromatin states: euchromatin and heterochromatin. Euchromatin is more accessible to *Sp*Cas9 compared with heterochromatin. Similarly, *Sp*Cas9’s efficiency in locating the PAM sequence (red bar) is higher on the linker than on the nucleosome. (B) Schematic representation of CRISPR/*Sp*Cas9 bound in the target site. The targeted strand (grey) pairs with the guide region (blue) of the sgRNA (orange). The cleavage is facilitated by the endonuclease HNH and RuvC domains, which cut the targeted and non-targeted strands, respectively. (C) Depiction of two primary DNA end types following *Sp*Cas9 cleavage. The blunt end (blue, on the right) is cleaved by both endonuclease domains just 3 nt upstream of the PAM. The 1 nt staggered end (pink, on the left) is cleaved 3 nt upstream of the PAM on the targeted strand and 4 nt upstream of the PAM on the non-targeted strand. DNA ends containing the PAM are termed PAM-proximal, while those without are termed PAM-distal. (D) After cleavage, the non-targeted strand may undergo post-cleavage processing via the activity of the RuvC domain. PAM-distal ends can be trimmed by up to 10 nt, while PAM-proximal ends can be blunted if a staggered end has been created by the primary RuvC cut. The extent of the post-cleavage activity is determined by the duration for which *Sp*Cas9 remains bound at the target site. (E) Overview of the four main pathways participating in *Sp*Cas9-induced double-strand break (DSB) repair. The selection of the repair pathway in the cell is strongly influenced by the cell cycle stage. The dominant pathway in the G_1_ and G_0_ phases is the classical non-homologous end joining (c-NHEJ). It results in either a perfect repair, a small deletion (commonly on the PAM-distal side; occurs mainly when the fourth nucleotide upstream of the PAM is G), or a 1 nt insertion (mostly duplication of the fourth nucleotide upstream from PAM, especially when the fourth nucleotide is A or T and the third nucleotide is G). During the S and G_2_ phases, DSBs can be repaired by the homologous recombination (HR), single-strand annealing (SSA), or polymerase-theta-mediated end joining (TMEJ) pathways. HR requires a sister chromatid or other template (grey), the 5' strands of DNA ends are resected, and the exposed 3' tail invades into the sister chromatid serving as a template for the repair (the scheme shows HR subcategory synthesis-dependent strand annealing; SDSA: see ‘Homologous recombination’ for more details). After the tail is elongated according to the homologous template, the invading strand is displaced and pairs with its complementary strand from the opposite side of the DSB; both 3' ends are elongated to refill the remaining gaps, and the resulting nicks are ligated. HR usually results in a perfect repair. SSA repair is mediated through direct repeats (long homology; LH) around the DSB. Similar to HR, the 5' ends of the DSB are resected, and thereafter the 3' tails hybridize together via the LH region. Then, 3' flaps are trimmed, followed by ligation of the remaining nicks, leading to larger deletions. The TMEJ pathway uses short (mostly 2–6 nt) microhomologies (MH, yellow) occurring near the DSB. In addition to the S and G_2_ phases of the cell cycle, it also occurs in the M phase. In TMEJ, the 5' ends are shortly resected, Polθ identifies MHs on opposite sides of the DSB, the 3' flaps are removed, and the remaining gaps beyond the 3' ends of the MH are filled in, followed by ligation of individual nicks. TMEJ results in small deletions.

The CRISPR/Cas technologies are built on the natural defence mechanisms of bacteria and archaea, which developed an RNA-mediated adaptive mechanism against invading viruses and plasmids (Garneau *et al.*, 2010^O^; Gasiunas *et al.*, 2012^O^;  Jinek *et al.*, 2012^O^). Yet, there are three types of CRISPR/Cas systems with a single effector protein: type II, V, and VI (Makarova *et al.*, 2020^O^). The most frequently used is CRISPR/Cas9 from *S. pyogenes* belonging to type II. *Sp*Cas9 is a large (~158 kDa) multidomain endonuclease that can be targeted to the DNA via a programmable 20 nt long 5' region of a chimeric single-guide RNA (sgRNA) called the ‘guide region’ (Fig. 1B; Jinek *et al.*, 2012^O^). The action of this two-component system of *Sp*Cas9 with loaded sgRNA, hereinafter abbreviated as *Sp*Cas9 for the whole ribonucleoprotein (RNP) for simplification, can be divided into several steps, which we describe in the following paragraphs: *Sp*Cas9 (i) searches for the PAM sequence, (ii) unwinds the DNA double helix while trying to hybridize the guide region with the target site, (iii) in the case of sufficient complementarity, *Sp*Cas9 cleaves the target sequence, and (iv) releases/is removed from the DNA cleavage products.

### Protospacer adjacent motif sequence recognition

Firstly, a Cas RNP searches for the PAM sequence, which in the case of *Sp*Cas9 is 5'-NGG-3' (where N represents any nucleotide; Gasiunas *et al.*, 2012^O^; Jinek *et al.*, 2012^O^; Karvelis *et al.*, 2015^P^) but other PAM sequences are recognized by engineered Cas9 or other Cas variants (see more in ‘Guide for using CRISPR for editing’). The PAM sequence is localized on the ‘non-targeted’ DNA strand (Fig. 1B) and is found by three-dimensional diffusion of *Sp*Cas9; that is, stochastic dynamic binding and unbinding (Sternberg *et al.*, 2014^O^). The two guanosines in the PAM sequence interact with two arginine residues of the *Sp*Cas9 (R1335 and R1333), which allows the melting of the DNA double helix and initiation of unwinding of the upstream sequence (Anders *et al.*, 2014^O^).

### Unwinding DNA and hybridizing single guide RNA with the target site

After the PAM sequence recognition on the non-targeted strand, the complex tries to gradually unwind the upstream DNA double helix and hybridize the 20 nt long guide region of the sgRNA with the ‘targeted strand’, gradually forming an R-loop structure (Fig. 1B). If complementarity is not found, the *Sp*Cas9 complex dissociates and searches for another PAM sequence (Sternberg *et al.*, 2014^O^). For stable binding to the region, only 9–10 matching nucleotides upstream from the PAM are needed, whereas longer but not complete complementarity is required for cleavage (Josephs *et al.*, 2015^O^; Singh *et al.*, 2016^O^).

### Cleaving the target site

#### Complementarity with single guide RNA

If there is complete (or sufficient) complementarity between the hybridized guide region and the target site, the HNH endonuclease domain swings inward and ‘locks’ *Sp*Cas9 in a conformation allowing cleavage. *Sp*Cas9 carries two endonuclease domains, HNH and RuvC. HNH cleaves the targeted strand and RuvC the non-targeted strand, resulting in a DSB (Sternberg *et al.*, 2015^O^; Raper *et al.*, 2018a^O^). The resulting DNA cleavage products can be classified as PAM-proximal (the end containing the PAM sequence) and PAM-distal (Fig. 1C).

#### Mismatches

Several studies showed that mismatches at 17–20 nt upstream from the PAM do not significantly reduce *Sp*Cas9 activity (measured as the mutagenesis efficacy; Josephs *et al.*, 2015^O^; Sternberg *et al.*, 2015^O^; Singh *et al.*, 2016^O^). On the other hand, the region 14–17 nt upstream from the PAM was shown to be extremely sensitive to mismatches as complementarity in this region is needed for the conformational change, which is necessary for the cleavage (Josephs *et al.*, 2015^O^; Sternberg *et al.*, 2015^O^; Raper *et al.*, 2018a^O^). These mismatch effects must be considered when selecting the target sequence to avoid any edits in unintended sites, so-called off-targets (further details are given later).

#### Blunt versus staggered end formation

When targeted to plasmids or relaxed DNA, it was initially reported that both domains cleave three nucleotides upstream from the PAM sequence, forming a blunt cut (Fig. 1C; Jinek *et al.*, 2012^O^; Sternberg *et al.*, 2014^O^). Later works contradicted this claim, and molecular dynamics simulations and *in vitro* assays showed that the preferred cleavage mode of *Sp*Cas9 produces staggered ends, with the RuvC domain cleaving four nucleotides upstream of the PAM, generating 1 nt 5' overhangs (Fig. 1C; Zuo and Liu, 2016^O^; Stephenson *et al.*, 2018^O^). Both types of cleavage products were observed or inferred *in vivo* in plant (Přibylová *et al.*, 2022^P^), yeast, and animal studies (Li *et al.*, 2015^O^; Lemos *et al.*, 2018^O^; Stephenson *et al.*, 2018^O^; Gisler *et al.*, 2019^O^).

#### Post-cleavage processing

Importantly, after the initial DNA cleavage, the *Sp*Cas9 RuvC domain can further trim the non-targeted strand at both PAM-distal and PAM-proximal DNA cleavage products. The PAM-distal fragment can be trimmed in the 3'–5' orientation up to a distance of 10 nucleotides. If the PAM-proximal fragment has a staggered end (which was generated by the cleavage of more than three nucleotides upstream of the PAM), the RuvC domain can blunt it. This post-cleavage trimming is very slow, so it may or may not occur depending on the time the *Sp*Cas9 complex stays bound on the DNA cleavage products (Fig. 1D; Stephenson *et al.*, 2018^O^; Aldag *et al.*, 2021^O^).

### Releasing the target site

After cleavage, *Sp*Cas9 remains tightly bound to both DNA cleavage products (by base-pairing with sgRNA and probably also by the PAM sequence binding) for a long time and is considered to be a single-turnover enzyme. The predicted release half-time differs within *in vitro* studies from several hours to several tens of hours (Sternberg *et al.*, 2014^O^; Gong *et al.*, 2018^O^; Raper *et al.*, 2018b^O^). Nevertheless, *in vivo* gene editing, which requires DNA cleavage, release, and subsequent repair, was detected already within the first hours after delivery of *Sp*Cas9 in the form of an RNP (van Overbeek *et al.*, 2016^O^). This suggests the existence of an efficient cellular mechanism for the ejection of *Sp*Cas9 from the cleavage products.

Nevertheless, release of the cleavage products from the *Sp*Cas9 complex *in vivo* remains poorly understood. There are many unresolved questions related to the dissociation rate, participation of various chromatin proteins, interactions with the transcription machinery, or the ability of *Sp*Cas9 to search for another target after ejection from the first site. The mechanism of *Sp*Cas9 release is also fundamental with regard to the character of the resulting genome edits because the fast release of cleavage products (e.g. due to triggering *Sp*Cas9 degradation; Sreekanth *et al.*, 2020^O^) can minimize their post-cleavage processing. Moreover, uncoupling from *Sp*Cas9 may be needed to recognize free DNA ends by the DNA repair machinery, although a fast attraction of the repair components might be triggered by the spontaneous release of the non-targeted ssDNA from the complex after the cleavage, which was observed *in vitro* (Aldag *et al.*, 2021^O^).

## Fundamentals of DNA repair

The cleavage of target DNA by *Sp*Cas9 creates a DSB. Cells are well equipped to detect and subsequently repair DSBs which naturally occur under stress and non-stress conditions. Efficient repair, necessary to maintain genome integrity, faces the challenge of meeting two conflicting requirements: the free DNA ends should be joined as quickly as possible, and the repair should optimally be error free. Reliably error-free repair requires a template molecule by which the broken DNA molecule can be repaired. However, such a template is only available on some occasions, depending mainly on the cell cycle phase.

The repair pathways can be divided into four major types based on the molecular mechanism and the machinery behind them (schemes of the pathways are shown in Fig. 1E). (i) Classical non-homologous end joining (c-NHEJ) directly joins free DNA ends and can work independently of any homology. This repair can be error free but generally is error prone due to frequent processing (mostly trimming) of DNA ends before ligation. (ii) Homologous recombination (HR) comprises several subcategories that all require the presence of a homologous template and are principally error free. (iii) Single-strand annealing (SSA) shares a lot of the molecular machinery with the HR pathway, but the end joining is mediated by the complementarity between long homologous regions (direct repeats) present at both DNA ends. Such joining always results in a deletion of one of the repeats and the sequence in between them. (iv) Polymerase theta-mediated end joining (TMEJ), also known as microhomology-mediated end joining (MMEJ) or alternative end joining (alt-EJ), joins the free DNA ends by a short complementarity between these ends, so consequently this pathway generates deletions which may also be coupled with insertions introduced by the polymerase theta (Polθ) activity. These different repair pathways have evolved to ensure optimal DSB repair under various circumstances and back each other up. Although it is not fully clear what exactly determines the choice of the repair pathway in a given situation, proteins that primarily detect DSBs may be involved in the decision.

The DSB is most probably recognized by either the KU70/KU80 heterodimer or POLY(ADP-RIBOSE) POLYMERASE 1 (PARP1). They both are highly abundant within the nucleus and were shown to reach the DSB site in <1 s (Yang *et al.*, 2018^O^). In *Arabidopsis thaliana*, KU70 and KU80 are expressed widely in most of the tissues and developmental stages in the dividing and non-dividing cells (Tamura *et al.*, 2002^P^; Yadav *et al.*, 2009^P^; Klepikova *et al.*, 2016^P^). In rice, it was reported in roots, leaves, internodes, meristems, and also in undifferentiated callus cells (Hong *et al.*, 2010^P^). In contrast, PARP1 is expressed specifically in tissues with actively dividing cells (Yadav *et al.*, 2009^P^; Klepikova *et al.*, 2016^P^). Both KU and PARP1 levels increase in response to the generation of DSBs (Doucet-Chabeaud *et al.*, 2001^P^; Tamura *et al.*, 2002^P^).

In human cell lines, PARP1 expression is repressed in the G_1_ and G_0_ phases of the cell cycle and is released at the end of G_1_, when the cell enters S phase. Moreover, PARP1 is an inhibitor of DNA-dependent protein kinase (DNA-PKcs), which is necessary for the c-NHEJ repair pathway in human cell lines (Pietrzak *et al.*, 2018^O^). However, for many years, no DNA-PKcs homologues were identified in many organisms. Recently, DNA-PKcs was identified in some *Viridiplantae* from green algae, bryophytes, to gymnosperms, but it was not found in angiosperms (Lees-Miller *et al.*, 2021^P^; Kumar, 2023^P^). The missing kinase indicates different regulations of c-NHEJ between organisms, so transferring the knowledge from one to another must be done with special care. An observation on a human cell line indicates possible down-regulation of c-NHEJ by phosphorylation of KU70 in the S, G_2_, and M phases by cyclin-dependent kinases. This phosphorylation happens in the region where KU70 interacts with KU80, and the authors showed that the KU70/KU80 heterodimer could not be formed when KU70 is phosphorylated at those sites (Mukherjee *et al.*, 2016^O^). In contrast, another study documented that PARP1 activity is even necessary for KU70/KU80 binding to the free DNA ends on some occasions (Caron *et al.*, 2019^O^). Besides those and other contradictory findings, it is essential to note that studies on animal cell lines might be misleading as the cell lines originate from different cancers and usually have impaired cell cycle control at some level, which is important because the cell cycle affects the occurrence and likely behaviour of the repair pathways.

### Classical non-homologous end joining

If the DSB ends are recognized by the KU70/KU80 heterodimer complex (Tamura *et al.*, 2002^P^), the DNA repair is usually mediated by the c-NHEJ pathway (with some exceptions described below). DNA ends protected by KU70/KU80 can be first processed (by exonuclease or DNA polymerase activity) or directly ligated by DNA LIGASE IV (LIG IV) with the help of its cofactor X-RAY REPAIR CROSS-COMPLEMENTING PROTEIN 4 (XRCC4) and other proteins (West *et al.*, 2000^P^).

The KU70/KU80 heterodimer is highly abundant in the nucleus and is well conserved among eukaryotes (West *et al.*, 2000^PO^; Pöggeler and Kück, 2006^PO^; Abbasi *et al.*, 2021^PO^). It forms a ring-like structure with a high affinity for various free DNA ends, where each end seems to be occupied by one heterodimer protecting it from extensive degradation (Walker *et al.*, 2001^O^; Britton *et al.*, 2013^O^; Chang *et al.*, 2017^O^; Zahid *et al.*, 2021^O^). The presence of KU70/KU80 was shown in all examined tissues of *A. thaliana*; however, the activity of the c-NHEJ pathway dominates mainly in the G_1_ and G_0_ phases of the cell cycle (Tamura *et al.*, 2002^P^).

### Homologous recombination

DSB repair by HR pathways relies on a large region of homology (several hundred nucleotides) between the broken DNA (adjacent to the DSB) and an intact template molecule. HR pathways occur within the S and G_2_ phases of the cell cycle when the sister chromatid is present and can be used as a template. Of all the pathways, HR is considered the only one which is essentially error free. The HR repair is initiated by a set of proteins that gradually bind or occur around the DNA ends: the MRN complex consisting of MEIOTIC RECOMBINATION 11 (MRE11; Hartung and Puchta, 1999^P^), RADIATION SENSITIVE 50 (RAD50; Gallego *et al.*, 2001^P^), and NIJMEGEN BREAKAGE SYNDROME 1 (NBS1; Akutsu *et al.*, 2007^P^) proteins; ATAXIA TELANGIECTASIA MUTATED (ATM; Culligan *et al.*, 2006^P^) kinase; COMPLETION OF MEIOTIC RECOMBINATION 1 (COM1; Uanschou *et al.*, 2007^P^); EXONUCLEASE1 (EXO1; Kazda *et al.*, 2012^P^); and a plant-specific transcription factor SUPPRESSOR OF GAMMA RESPONSE 1 (SOG1; Culligan *et al.*, 2006^P^; Yoshiyama *et al.*, 2013^P^).

Based on the groundwork done mostly on yeast and animals, the whole HR pathway can be divided into several steps, which seem to be shared with plants since most players and mechanisms were shown to be conserved among these groups of organisms: (i) recognition of the DSB; (ii) resection of DNA ends by nucleolytic activities (i.e. generation of long 3ʹ ssDNA tails) and formation of a nucleoprotein filament; (iii) search for homology and invasion of one or both 3ʹ strands into the template molecule; (iv) extension of the invading strand(s); (v) release of the extended 3ʹ DNA tail(s) and joining the free DNA ends by annealing of their complementary elongated 3ʹ tails; and (vi) refilling the remaining single-stranded gaps and ligation.

#### Detailed description of each step of the HR pathway

(i) There is still some uncertainty concerning the recognition of a DSB that would direct the repair pathway to HR. There are the two above-mentioned leading candidates for the potential sensors of DSBs, PAPR1 and the KU70/KU80 complex, but none has been directly dedicated to the HR pathway (Yang *et al.*, 2018^O^).
(ii) The resection of DNA ends is mediated primarily by the MRN complex. The proposed mechanism involves two MRE11 with two RAD50 that form a heterotetramer (M_2_R_2_), which binds to the DNA ends via the DNA-binding domains of RAD50 and bridges the two broken DNA ends via its coiled-coil domains. MRE11 interacts with NBS1, which controls the activity of the whole MRN complex (M_2_R_2_N_2_) and recruits ATM kinases (Gallego *et al.*, 2001^P^; Daoudal-Cotterell *et al.*, 2002^P^; Hopfner *et al.*, 2002^O^; Akutsu *et al.*, 2007^P^; Williams *et al.*, 2009^O^). ATM activates itself by self-phosphorylation and subsequently phosphorylates many other proteins such as MRE11, NBS1, COM1 (Penkner *et al.*, 2007^O^; Uanschou *et al.*, 2007^P^), transcription factor SOG1 (Culligan *et al.*, 2006^P^; Yoshiyama *et al.*, 2013^P^), or histone H2AX, which together lead to the cell cycle arrest and proceeding to DNA repair or, in the case of extreme DNA damage, to cell death (Lavin *et al.*, 2005^O^; Amiard *et al.*, 2010^P^; Shen *et al.*, 2021^P^).
  When COM1 is activated through ATM signalling, it interacts with RAD50, promoting its ATPase activity, resulting in the activation of the MRE11 endonuclease activity (based on yeast studies; Cannavo and Cejka, 2014^O^; Reginato *et al.*, 2017^O^). MRE11 nicks the DNA strands upstream from the 5' termini (yeast Mre11 nicks naked DNA up to 300 nt from the ends; Garcia *et al.*, 2011^O^) and resects both DSB ends by subsequent 3'–5' exonucleolytic degradation of the 5' strands towards the DSB (while possibly removing proteins protecting the DNA ends; Myler *et al.*, 2017^O^). In the opposite direction from the nick, the DNA ends are further resected in the 5'–3' orientation, primarily by EXO1 (Mimitou and Symington, 2008^O^; Gong *et al.*, 2017^O^). Such bidirectional resection of DNA exposes long 3' ssDNA strands (Garcia *et al.*, 2011^O^; Syed and Tainer, 2018^O^; Tisi *et al.*, 2020^O^).
  Long exposed 3' ssDNA overhangs are immediately protected by RPA complexes, each consisting of three REPLICATION PROTEIN A (RPA) subunits (Aklilu *et al.*, 2014^P^). With the help of BREAST CANCER 2 LIKE 2A (BRCA2; Mayer *et al.*, 1999^P^; Dumont *et al.*, 2011^P^), the RPA complexes are subsequently exchanged by RAD51 (Doutriaux *et al.*, 1998^P^; Dray *et al.*, 2006^P^) and RAD54 (Osakabe *et al.*, 2006^P^), forming nucleoprotein filaments (Osakabe *et al.*, 2006^P^; Hirakawa *et al.*, 2017^P^; Syed and Tainer, 2018^O^; Tisi *et al.*, 2020^O^).
(iii) The nucleoprotein filament searches for a homologous DNA sequence, ideally a sister chromatid. However, other homologous molecules may also be used as a template, such as artificially inserted oligonucleotides, which are used in gene editing in combination with the CRISPR/Cas system (Fauser *et al.*, 2014^P^; see ‘Guide for using CRISPR for editing’). The nucleoprotein filament of one or both DSB ends invades into the template molecule and anneals with the complementary strand(s), forming either a simple displacement-loop (D-loop) in the synthesis-dependent strand annealing (SDSA) pathway or a double D-loop in the double-strand break repair (DSBR; Roth *et al.*, 2012^P^) pathway, respectively.
(iv–vi) The invaded filament (or filaments) is then extended by DNA polymerase (in yeast dominantly by Polδ; Maloisel *et al.*, 2008^O^) according to the template. This is a crucial step to restore the sequence potentially lost at the DSB site. In the SDSA pathway, the single 3ʹ DNA tail in the D-loop is extended and then displaced from the template. The elongated tail is used to anneal with the 3' tail from the opposite side of the break to bridge the DSB. Then, both 3' ends are elongated to refill the remaining ssDNA gaps and ligated. In the DSBR pathway, the double D-loop results in the double Holliday junction (Roth *et al.*, 2012^P^).

### Single-strand annealing

The SSA repair pathway seems to share the same initial steps with the previously described HR until the formation of long 3' tails (generated by joint activity of MRE11 and EXO1) and their coverage by RPA proteins. Unlike HR, the 3' tails are not covered by RAD51 or RAD54, but RAD52 is involved, and the tails do not search for a homologous sequence at a parallel template molecule, but rather they search each other. If sufficiently long complementarity (a direct repeat several tens to a few hundred nucleotides long) is present near both sides of the DSB, the strands can anneal, the residual 3' ssDNA flaps are removed, and the nicks are ligated (Roth *et al.*, 2012^P^; Bhargava *et al.*, 2016^O^; Vu *et al.*, 2022^P^). As the homology is found within two fragments of originally the same DNA molecule, the DNA region between the two repeats and one repeat is lost.

### Polymerase-theta-mediated end joining

The initiation of the TMEJ repair pathway is believed to start with recognition of the DSB via PARP1 (Chen *et al.*, 1994^P^; Doucet-Chabeaud *et al.*, 2001^P^; Jia *et al.*, 2013^P^). PARP1 is a broadly conserved protein across eukaryotes (Citarelli *et al.*, 2010^PO^; Song *et al.*, 2015^PO^) that searches for the DNA break via a rapid ‘monkey bar’ mechanism (Rudolph *et al.*, 2018^O^). It specifically binds to both single- and double-strand DNA breaks. After binding to the break, it poly(ADP)-ribosylates (PARylates) itself, DNA ends, histones, and other acceptor proteins (Taipakova *et al.*, 2020^P^; van Beek *et al.*, 2021^O^). PARP1, together with PARP2, recruits many proteins from the DNA repair machinery, including the MRN complex (Haince *et al.*, 2008^O^), Polθ—a central and, in higher eukaryotes, widely conserved component of the TMEJ pathway (Harris *et al.*, 1996^O^; Inagaki *et al.*, 2006^P^; Chan *et al.*, 2010^O^; Mateos-Gomez *et al.*, 2015^O^; Schaub *et al.*, 2022^O^)—and directly or indirectly also other proteins such as DNA LIGASE III (LIG3), X-RAY REPAIR CROSS-COMPLEMENTING GROUP 1 (XRCC1), or LIG1 (Mosler *et al.*, 2022^O^).  TMEJ was reported to be active in the S and G_2_ phases of the cell cycle (Sfeir and Symington, 2015^O^; Dutta *et al.*, 2017^O^), and a recent study on human cell lines showed that TMEJ is specifically activated in the M phase when c-NHEJ and HR pathways are attenuated (Brambati *et al.*, 2023^O^).

After the recognition of the DSB by PARP1, the MRN complex resects the 5' DNA strands, probably in a similar manner to the case of HR, but EXO1 is not recruited to the 5' termini of the nick, leading to the formation of a relatively short 3' ssDNA overhang (Sfeir and Symington, 2015^O^). Polθ binds to these 3' ssDNA tails and searches for short (usually 2–6 bp) microhomologies, primarily on the opposite 3' DNA tail if it is available. During this process, it can remove RPA complexes if they coat the ssDNA tails (Schaub *et al.*, 2022^O^). Upon finding the microhomology, any free 3' flaps are removed by 3' exonucleases such as ERCC1-RAD1 (Dubest *et al.*, 2002^P^), and then the DNA is elongated from the site of the microhomology up to the end of the gap (Hogg *et al.*, 2012^O^).  Thereafter, the DNA is sealed with XRCCA/LIG3 or LIG1 (West *et al.*, 2000^P^; Liang *et al.*, 2008^O^).

In cases when Polθ interacts with a DNA molecule other than the opposite DSB tail, its activity results in a short deletion combined with a templated insertion originating from the ‘other’ donor molecule. Such a mechanism is involved, for example, in *Agrobacterium* T-DNA integration (van Kregten *et al.*, 2016^P^; van Tol *et al.*, 2022^P^) or in the incorporation of single-stranded oligodeoxynucleotides (Ferenczi *et al.*, 2021^P^).

## Factors affecting the overall efficacy and outcome of CRISPR/*Sp*Cas9 mutagenesis

The *Sp*Cas9 activity and the subsequent DSB repair are affected by many factors, which all contribute to the resulting nature and frequency of genome edits. According to the mechanism of *Sp*Cas9, the first factor is the accessibility of the PAM sequence, whether it is localized in the tightly condensed heterochromatin or in various types of more accessible loosened euchromatin (Fig. 1A). Once *Sp*Cas9 is in the proximity of the target site, its binding depends on whether the PAM is located on DNA wrapped around the nucleosome or the linker DNA. Next, it depends on the sequence context of the target site and how easily DNA strands are dissociated and the targeted strand hybridized with the sgRNA. After the cleavage, the character and frequency of individual edits depend on how quickly *Sp*Cas9 is removed from the target site and which DNA repair pathway is available, which is greatly affected by the cell cycle phase.

The effects of various factors on Cas9 editing were mostly assessed by analysing the mutagenesis outcomes *in vivo*. Since some factors can influence several steps of the process (e.g. the presence of the target site in heterochromatin can affect both the efficiency of DSB formation and the subsequent DSB repair), it is hard to assess the impact of some factors on each step of the editing process separately. Moreover, one should consider that higher activity of the error-free repair by HR (and partly also by c-NHEJ) usually cannot be distinguished from lower efficacy of *Sp*Cas9 cleavage, and vice versa. Keeping this in mind, we summarize some of the factors affecting the overall *Sp*Cas9 efficacy and mutagenesis outcomes below.

### Chromatin state

#### Finding the target

It was reported in *A. thaliana* that when targeting the identical sequence in the euchromatin and heterochromatin region, the efficacy in the heterochromatin was lower. The authors linked certain chromatin features with higher and lower Cas9 mutagenesis. For example, activation histone marks such as acetylation, H3K36me3, and H3K4 methylation were linked with the highest mutagenesis efficacy. On the other hand, heterochromatin features connected with the presence of H2A.W, H3K9me1, H3K9me2, H3K27me1, and DNA hypermethylation were associated with low efficacy (Weiss *et al.*, 2022^P^). In *Nicotiana benthamiana* leaves, we showed that introducing high levels of DNA methylation into the promoter of a reporter transgene caused a decrease in the efficacy for seven out of eight closely located targeted sites, as expected, but the remaining target showed completely the opposite trend, indicating that the relationship is not so straightforward as previously supposed. Moreover, the introduction of DNA methylation into the gene body of the transgene had no significant effect on the mutagenesis efficacy (Přibylová *et al.*, 2022^P^).

Importantly, many works in several plant species and other organisms reported successful *Sp*Cas9 mutagenesis of targets in both chromatin states (Yu *et al.*, 2013^O^; Chen *et al.*, 2016^O^; Feng *et al.*, 2016^P^; Daer *et al.*, 2017^O^, 2018, Preprint^O^; Kallimasioti-Pazi *et al.*, 2018^O^; Weiss *et al.*, 2022^P^). In general, these studies show that editing in the heterochromatin region tends to be less effective but possible (Fig. 1A).

#### *Releasing* Sp*Cas9 from the cleavage products*

Apart from getting to the target, the mutation outputs are affected by the time *Sp*Cas9 spends on the target site after the cleavage because of the 5'–3' and 3'–5' post-cleavage activities of the RuvC domain (Stephenson *et al.*, 2018^O^). *Sp*Cas9 is considered to be a single-turnover enzyme. When bound *in vitro*, pulling the PAM-proximal DNA end out of the complex requires a concentration of urea as high as 7 M (Sternberg *et al.*, 2014^O^; Gong *et al.*, 2018^O^) or a temperature higher than 80 °C, which leads to complete dissociation of *Sp*Cas9 from the cleavage products. In contrast, at 37 °C, only ~10% of the DNA was released from the complex (David *et al.*, 2022^O^). The question is: how is *Sp*Cas9 detached from its target *in vivo*, as dissociation of the heteroduplex formed by the guide region of sgRNA and the targeted DNA strand requires a lot of energy? Artificially, this can be achieved by inducing *Sp*Cas9 degradation (Sreekanth *et al.*, 2020^O^). The presence and activity of factors naturally promoting the detachment of the *Sp*Cas9 from its target probably differ in euchromatin, heterochromatin, regulatory regions of promoters, transcriptionally active regions, untranscribed regions, etc. Moreover, these factors probably also affect the subsequent DNA repair pathways. Not much is known about these factors and mechanisms, and future studies are needed.

### PAM sequence recognition

#### Spatial availability of PAM

*In vitro* and *in vivo* studies on human cell lines and yeast indicate that the spatial availability of the PAM sequence can affect the *Sp*Cas9 mutagenesis efficacy. It is higher when the PAM is located at linker DNA or in a region with low nucleosome occupancy compared with the efficacy when the PAM is located on DNA wrapped on the nucleosome core or in a region with high nucleosome occupancy (Fig. 1A; Hinz *et al.*, 2015^O^; Horlbeck *et al.*, 2016^O^; Yarrington *et al.*, 2018^O^). We can also speculate about the effect of the PAM sequence facing outwards or inwards of the nucleosome, which can complicate *Sp*Cas9 binding to such a spatially ‘hidden’ PAM. Nevertheless, even if there is such an effect, it is almost impossible to consider it when selecting the target sites because the position of nucleosomes *in vivo* are mostly unknown, they are dynamic structures, and usually only nucleosome positions around the transcription start sites are more conserved (Liu *et al.*, 2015^P^).

#### PAM sequence context

It was shown that the immediate sequence context surrounding the GG of the PAM sequence affects the recognition of the target site and, subsequently, the overall mutagenesis efficacy. In human cell lines, Gorodkin’s lab compared all possible combinations of PAM with one additional downstream nucleotide (5'-NGGN-3') at multiple target sites. Their results showed that the 5'-GGGH-3' context (where H represents A, T, or C) had the highest efficacy, whereas 5'-HGGG-3' had the lowest (Corsi *et al.*, 2022^O^).

#### Non-canonical PAM sequence recognition

*Sp*Cas9 recognizes not only the canonical 5'-NGG-3' but also non-canonical PAMs, as shown in a large-scale *in vitro* study on *Sp*Cas9 without endonuclease activity (Boyle *et al.*, 2017^O^). Identical target sequences with 5'-NAG-3' PAMs were mutated in rice with high efficacy, reaching 50–105% of the efficacy in the target sites with the canonical PAMs (Meng *et al.*, 2018^P^). Later, a complex study on an animal cell line showed that other PAM variants could also be recognized, with 5'-NAG-3' and 5'-NGA-3' having the highest median efficacy, which reached 30% and 20% of the median efficacy of the canonical 5'-NGG-3'. Additionally, this efficacy was the highest within the individual groups when the N was represented by G (Corsi *et al.*, 2022^O^).

### Target site sequence

After recognizing the PAM sequence, *Sp*Cas9 unwinds the target strand. Specific nucleotides at certain positions within the target region affect efficiency and can also be used to predict mutation outcomes. In addition to the intended perfectly matching on-targets, *Sp*Cas9 can also hybridize with targets that differ in the number of mismatched nucleotides and their position, which affects the efficiency of mutagenesis and mutation results in various ways (Sternberg *et al.*, 2014^O^; Josephs *et al.*, 2015^O^; Raper *et al.*, 2018a^O^).

#### Mismatches

As described in ‘Cleaving the target site’, *Sp*Cas9 can bind not only to target sequences that perfectly match with sgRNA but also to sites matching perfectly only with the 3' region of the sgRNA (‘seed sequence’ of 8–12 nt proximal to the PAM; Sternberg *et al.*, 2014^O^).  This plasticity in the Cas9 target recognition allows the editing of two or more closely related paralogous genes by one sgRNA. However, unrelated and unwanted loci, known as off-targets, can also be affected when they share sufficient sequence similarity.  The efficacy of cleavage is influenced by the positions of mismatches. In certain off-target sites, *Sp*Cas9 merely binds without inducing cleavage, while at other sites it actively cleaves (Josephs *et al.*, 2015^O^; Sternberg *et al.*, 2015^O^; Singh *et al.*, 2016^O^; Raper *et al.*, 2018b^O^). Thereby, the careful selection of the target site is crucial, with a focus on minimizing any potential off-targets or their negative effects within the genome when they are unavoidable.

For this purpose, numerous tools have been developed to identify and assess potential off-targets and their overlap with various genomic elements, such as exons, introns, etc.  Typically, the user inputs the intended 20 nt target sequence and the organism (species name) of interest. In situations where the organism’s genome is unavailable, it is advisable to use the closest relative, but then the prediction value is limited. For plants, several online tools can be used; for example, CRISPR-P 2.0 (when this review was submitted, this included 49 plant genomes; Liu *et al.*, 2017^P^), which is plant oriented, and its sub-versions also specialize in crops (He *et al.*, 2021^P^); Cas-OFFinder (>220 plant genomes; Bae *et al.*, 2014^PO^), which is regularly updated and in addition to plants also contains other organisms such as vertebrates, insects etc.; CRISPOR (Concordet and Haeussler, 2018^PO^); and CCTop (Stemmer *et al.*, 2015^PO^). By default, most tools consider off-targets only in the context of the canonical 5'-NGG-3' PAM sequence. Since *Sp*Cas9 also recognizes other PAMs, such as 5'-NAG-3' and 5'-NGA-3', relatively efficiently (Boyle *et al.*, 2017^P^; see ‘PAM sequence recognition’), dealing with such off-targets is also necessary.

If the potential ‘harmful’ off-targets cannot be avoided, it is advisable to reduce the longevity of *Sp*Cas9 (see ‘How to deliver and get rid of the *Sp*Cas9 and sgRNA’) or minimize the efficacy on off-targets by shortening the guide region sequence. It was shown in human cells that if the guide region of the sgRNA is only 17 nt long, the complex still efficiently cuts the target sequence, but it is very sensitive to mismatches (Fu *et al.*, 2014^O^; J.-P. Zhang *et al.*, 2016^O^). Such truncated guide region sequences have also been successfully used in Arabidopsis (Osakabe *et al.*, 2016^P^). However, more extensive studies comparing the effect of shortened guides on- and off-targets in plants are lacking.

#### Sequence effect

The sequence of the target site itself and its surroundings strongly affect the overall mutagenesis efficacy and mutation outputs at two main levels. The first level includes factors affecting the energy balance of the strand separation in the target site and hybridization of the guide region of sgRNA. It includes the energy needed to linearize the possible secondary structures on the guide region, the energy of the initial melting, and the energy balance throughout the full-length melting/hybridization process. It was shown that guide regions harbouring G as the first nucleotide upstream from the PAM greatly outperformed the other nucleotides at the same position in overall mutation efficacy. The second strongest effect was observed at the third position upstream from the PAM, favouring C (Corsi *et al.*, 2022^O^).

At the second level, the sequence surrounding the cleavage site can subsequently affect DNA repair. Some works showed that certain nucleotide contexts of the guide region pre-determine the mutation output (van Overbeek *et al.*, 2016^O^; Chen *et al.*, 2019^O^). For example, it was shown in human cell lines that if the fourth nucleotide upstream from the PAM, on the non-targeted strand, is A or T in combination with G in the third position, there is a high chance that the mutation output will be a single-nucleotide insertion. Alternatively, if the fourth nucleotide is G, the likeliness of the insertion is very low, and most of the mutation outputs will be deletions (Taheri-Ghahfarokhi *et al.*, 2018^O^; Chen *et al.*, 2019^O^; Fu *et al.*, 2021^O^), which was also observed in plants (Bennett *et al.*, 2020^O^; Přibylová *et al.*, 2022^P^). As the single-nucleotide insertions are mostly duplications of the fourth nucleotide, mainly in cases when the fourth nucleotide is A or T, these events may be explained by the tendency of *Sp*Cas9 to form 1 nt staggered ends which are filled in by polymerase activity (Fig. 1E). Nevertheless, the c-NHEJ pathway must be available to make such a single-nucleotide insertion, indicating that these mutation outcomes are also cell cycle dependent.

### Cell cycle stage and the availability of DNA repair mechanisms

The choice of repair pathway seems to be accurately regulated during the cell cycle, so it is important to know at what stage of the cell cycle *Sp*Cas9 forms the DSB and/or when the complex is displaced from the target site to allow the repair. In G_1_ and differentiated cells in the G_0_ phase, the c-NHEJ pathway predominates because it can be error free, and cells cannot use the safest HR pathway because the sister chromatid is unavailable as a template. From the beginning of S phase (until the end of G_2_ phase), chromosome segments that have undergone replication can be repaired by the error-free HR. However, if a DSB occurs in a region where DNA has not yet been replicated, the break must be quickly sealed before the replication fork appears. On those occasions, c-NHEJ, TMEJ, and SSA may play their roles. Finally, TMEJ seems to be specifically activated in M phase when c-NHEJ and HR pathways are attenuated (Brambati *et al.*, 2023^O^). The schematics of the different DNA repair pathways and their dependence on the cell cycle are shown in Fig. 1E. Considering this cell cycle effect, the *Sp*Cas9 mutation outcome can significantly differ between meristems (proliferating cell cultures) and differentiated cells, so findings obtained with one model cannot be simply transferred to the other.

#### c-NHEJ repair results mostly in single-nucleotide insertions and short PAM-distal deletions

The c-NHEJ pathway is the dominant pathway in the G_1_ and G_0_ phases of the cell cycle (Fig. 1E). After the *Sp*Cas9 cleavage followed by early release, unprocessed DNA ends might be directly re-ligated, resulting in no mutation. However, in differentiated cells, *Sp*Cas9 cleavage often results in short deletions, which occur specifically on the PAM-distal side of the cleavage (Přibylová *et al.*, 2022^P^), probably due to the post-cleavage 3'–5' exonuclease activity of the RuvC domain. These deletions are significantly enriched for those in which the end joining may have been stabilized by the complementarity between the 5'-terminal nucleotide of the PAM-proximal end and a complementary nucleotide at the exposed 5' overhang of the PAM-distal end (Přibylová *et al.*, 2022^P^). After the staggered cleavage, the 5' overhangs (mostly 1 nt long) can be filled in by polymerase activity before the ligation. That results mostly in the insertion/duplication of the overhanging fourth nucleotide upstream of the PAM. Whether the nucleotide will be duplicated or not strongly depends on the nucleotides present at the third and fourth position upstream of PAM, as described in ‘Sequence effect’. Usually, the single-nucleotide insertions are the most frequent mutation outputs when working with differentiated leaf cells or protoplasts prepared from these cells (Ueta *et al.*, 2017^P^; Hudzieczek *et al.*, 2019^P^; Raitskin *et al.*, 2019^P^; Přibylová *et al.*, 2022^P^).

#### TMEJ repair: short deletions

The TMEJ pathway is the most error-prone pathway working within the S, G_2_, and M phases of the cell cycle (Fig. 1E). DSBs repaired by the TMEJ pathway result mostly in short deletions extending to both sides of the cleavage site. These deletions are flanked by microhomologies that are crucial for TMEJ. Thus, the occurrence of a short (minimally 2–6 nt long) direct repeat present at both sides of the cleavage position significantly increases the frequency of deletions that cover one of the repeats and the sequence in between. This characteristic outcome of TMEJ repair is ‘typical’ for *Sp*Cas9 mutagenesis in proliferating animal cells (Bae *et al.*, 2014^O^; van Overbeek *et al.*, 2016^O^; Kurgan *et al.*, 2021^O^). Importantly, based on our data analysis, possible TMEJ footprints (short deleted regions surrounded by microhomologies) also occur in *Sp*Cas9 edits performed in *Chlamydomonas reinhardtii* (Baek *et al.*, 2016^P^) or in young developing leaves of *N. benthamiana* (Ellison *et al.*, 2020^P^).

#### HR and SSA

HR with an externally provided template can lead to precise pre-programmed edits, which is the ‘holy grail’ for genome editing. However, without the template, HR mostly results in error-free DNA repair by using the sister chromatid. Perfect repair is undesired in genome editing, and, generally, it is not possible to distinguish whether unmutated target sites were repaired without errors or were not cleaved at all. The SSA repair pathway, which shares the initial steps with HR, requires the presence of longer direct repeats at both sides of the cutting point. Therefore, it is restricted to specific genome locations with these sequence characteristics where it can be relatively frequent (Orel *et al.*, 2003^P^).

### Other indirect factors

There are also other factors affecting the *Sp*Cas9 mutagenesis efficacy. In general, any greater stress can play a role. On *N. benthamiana*, we have reported that infecting plants with a wild-type tobacco rattle virus increased the overall mutagenesis efficacy without changing the ratio of mutation output categories (ratio of insertions/deletions and representation of specific deletions). A similar effect was achieved by using a higher concentration of *Agrobacterium* for transformation (Přibylová *et al.*, 2022^P^). Several works showed an increase of the mutagenesis efficacy by increasing the cultivation temperature (LeBlanc *et al.*, 2018^P^; Milner *et al.*, 2020^P^; Blomme *et al.*, 2022^P^). The first explanation offered is that plants under stress favour faster and energetically less demanding repair pathways than the error-free HR. However, some of these studies were done on differentiated cells in G_0_, so either the error rate of the c-NHEJ pathway was increased under stress conditions, or the activity of *Sp*Cas9 was higher, for example due to chromatin loosening. It was also shown in animal cell lines that specifically modified *Sp*Cas9 can be efficiently targeted for proteasomal degradation by small artificial molecules. Shortening the operating life affected the choice of DNA repair pathway and significantly improved the ratio between target and non-target edits (Sreekanth *et al.*, 2020^O^).

## Guide for using CRISPR for editing

Based on the information we have described in previous sections, an imaginary decision tree may have started to form in the reader’s mind—how to select a suitable target site and design the sgRNA or how to design the experimental set-up to increase the probability of obtaining their desired mutation output. Going through the following list of instructions/questions might help to achieve the goal. In all cases, the suitable target or targets in the region of interest can be found *in silico* using one of the available tools such as CRISPR-P 2.0 (Liu *et al.*, 2017^P^), CRISPR-Local (Sun *et al.*, 2019^P^), CRISPOR (Concordet and Haeussler, 2018^PO^), or CRISPR-PLANT (Xie *et al.*, 2014^P^). Generally, targets with the PAM sequence 5'-GGG-3' and with G at the first position upstream of the PAM will probably be cleaved more efficiently (Farboud *et al.*, 2019^O^). Importantly, evaluation of off-targets for individual candidate target sites is highly advisable. In addition to the number and predicted efficiency of cleavage in the off-targets, their position in the genome should also be considered. The lowest risk of affecting the phenotype due to mutation in the off-target is connected with intergenic regions, whereas genic regions should be avoided. However, even cutting in multiple ‘safe’ targets can lead to chromosome rearrangements (Rönspies *et al.*, 2022a, b^P^).

### What is the desired type/goal of the mutation?

We have categorized the editing events into six categories; the first three are divided according to their effect, and the remaining three are according to the (advanced) mechanism of the editing. Consequently, the categories are not mutually exclusive.

#### Knockout mutations, short deletions, and insertions

*Sp*Cas9 mutagenesis is mostly used for gene knockouts. In such a case, the aim is to cause a frameshift in the gene, which is deleterious for the protein function. For that, targeting *Sp*Cas9 to a region coding for an important protein domain or to the 5' region of the coding sequence is the best (but not too close to the start codon to avoid activating an alternative start codon). The frameshift can be achieved by single-nucleotide insertion or a deletion whose length is not divisible by three. Since the editing efficacy by a single selected sgRNA can be low, it is advisable to use multiple sgRNAs per gene. On the other hand, it is sometimes desirable to create deletions to remove one or more amino acids, which can, for example, remove the target site for an miRNA while preserving the protein’s function. In this case, it is advantageous to choose the cleavage site so that there is microhomology in the vicinity to ensure in-frame deletion (Ferreira and Reis, 2023^P^).

When insertions are the goal, the fourth nucleotide upstream from the PAM on the guide region of the sgRNA should be optimally A or T in combination with G on the third position (if the repair is done by c-NHEJ). When using the egg cell-specific promoter or working with proliferating cell culture, cutting between two short, direct repeats (2–6 bp) in close proximity frequently results in a deletion of one repeat and the sequence between them (repair done by TMEJ). Those relatively short mutations can be quickly detected by T7 assay, specific primers, or Sanger sequencing (for the analysis, we recommend using the TIDE tool; Brinkman *et al.*, 2014^O^).

#### Longer deletions with or without a frameshift

*Sp*Cas9 supplemented with two sgRNAs can be used to induce a deletion in between their target sites. On human cell lines, it was shown that the most efficient was using two targets with PAMs oriented outwards from the intended deletion (Bothmer *et al.*, 2017^O^). As the cutting sites are predominantly located between the third and the fourth nucleotide upstream of the PAM sequence, suitable sgRNA targets can be selected to either shift (causing a knockout) or preserve the reading frame. The latter option can be used, for example, to specifically remove a gene region coding for a domain whose function is the subject of interest. Longer deletions are also suitable for functional analysis of *cis*-regulatory elements in a promoter region. Moreover, the larger knockout deletions are very easy to genotype since only the truncated PCR fragment needs to be searched on electrophoresis. Deletions induced by a *Sp*Cas9 supplemented with a pair of sgRNAs reached the size of up to several hundred kilobase pairs in rice (Zhou *et al.*, 2014^P^) and up to 65 Mbp in human cell lines (Eleveld *et al.*, 2021^O^). However, while some works reported that it worked nicely (Čermák *et al.*, 2017^P^; Srivastava *et al.*, 2017^P^; Henderson *et al.*, 2020^P^; Beying *et al.*, 2021^P^), some other users reported that in their system, larger deletions were very rare and most of the mutations were single-nucleotide insertions within both target regions.

#### Chromosomal rearrangements

Introducing two DSBs on the same chromosome can also result in an inversion of the region within the brakes (Siebert and Puchta, 2002^P^). It seems that there is no limit to the size of the inverted region, and it is also possible to make translocations/substitutions of whole chromosomal fragments when the two DSBs occur on different chromosomes (Gehrke *et al.*, 2022^P^). Such a large heritable reciprocal translocation was recently done in *A. thaliana* using *Sa*Cas9 (Beying *et al.*, 2021^P^). For more information, we recommend reading the protocol from Holger Puchta’s lab (Rönspies *et al.*, 2022a^P^).

#### Various pre-designed edits by homology-directed repair (including substitutions and pre-designed insertions of short or long nucleotide sequences)

These edits can be introduced by homology-directed repair (HDR) of the cleaved target when an exogenously provided oligonucleotide is used as a template molecule. This type of HDR is often called oligonucleotide-directed mutagenesis (ODM). The donor oligonucleotide template must have homologous arms that are adjacent to the modified/inserted region. The central region either can contain a modified sequence of the target site (insertion, deletions, substitutions, and combination of all) or a long transgene can even be incorporated by this approach (Ferenczi *et al.*, 2021^P^; Chen *et al.*, 2022^P^).

In *Caenorhabditis elegans*, it was shown that for induction of point mutations (substitutions or short insertions/deletions) in close proximity to the *Sp*Cas9 cut site (up to 30 bp), the delivery of ssDNA as a donor template is more efficient than using dsDNA. Moreover, the efficacy also depended on the donor strand orientation; ssDNA should be complementary to the targeted strand for mutations upstream from the cut site (on the PAM-distal DNA end), ssDNA complementary to the non-targeted strand is suitable for mutations downstream from the cut site. In *C. elegans*, for longer insertions, dsDNA as a template was shown to be best in combination with using two Cas9 target sites (Farboud *et al.*, 2019^O^).

Since the machinery of HDR pathways is active in the S and G_2_ phases of the cell cycle, the edited cells should undergo cell divisions. It was shown that in human cell lines, increasing the number of cells in the S and G_2_ phases enhanced the frequency of HDR (Yang *et al.*, 2016^O^). For more details, we recommend reading a recent plant update about genome editing by HDR from Xia’s lab (Chen *et al.*, 2022^P^).

#### Base editing by enzymes fused to nCas9

Partially inactivated *Sp*Cas9 variants which can cut only one DNA strand (nicking) due to a mutation in one of the two nuclease domains (Cong *et al.*, 2013^O^; Ran *et al.*, 2013^O^; Barrangou and Marraffini, 2014^O^) can be used to introduce specific mutations at the cleavage site. The ‘nickase’ SpCas9 mutated in the RuvC domain (nCas9 D10A) or dead Cas9 (dCas9), which has mutations in both nuclease domains (mutations D10A and H840A) in fusion with a nucleotide base deaminase, can be used for generating single or a few base exchanges near the target site. Most frequently, cytosine base editors (CBEs), which convert C to T, and adenine base editors (ABEs), converting A to G, are used for editing (Komor *et al.*, 2016^O^; Gaudelli *et al.*, 2017^O^). When using the nCas9, the nickase activity enhances the base editing efficiency compared with dCas9. Both types of these base editors were successfully used in different plant species (Bharat *et al.*, 2020^P^).

#### Templated edits by reverse transcriptase fused to nCas9 (prime editing)

In theory, prime editing can be used to achieve specific short insertions, deletions, and substitutions, but long insertions, chromosomal translocations, and inversions are also possible, as shown in non-plant model systems. The first prime editing version consisted of the ‘nickase’ *Sp*Cas9 with a mutation in the HNH domain (nCas9 H840A) fused with a reverse transcriptase, supplemented with modified sgRNA—prime editing guide RNA (pegRNA). The pegRNA guides nCas9 to the target via the ‘traditional’ 5' guide region, but it also carries a segment extending the 3' end, which is used by the reverse transcriptase as a template for the desired edit (Anzalone *et al.*, 2019^O^). Subsequently, several enhanced versions were developed to increase the overall efficacy (Nelson *et al.*, 2022^O^). However, all versions have been developed mainly on human cell lines, and the effective application in plants encounters many obstacles, especially in dicots.  The efficiency varies from a few edits per million to the higher tens of percent. The most recent information about prime editing in plants is summarized in a recent review from Vu *et al.* (2024^P^).

### How to deliver and get rid of the *Sp*Cas9 and sgRNA

(i) Most frequently, *Sp*Cas9/sgRNA is produced from a transgenic cassette integrated into the genome. It is usually introduced by some of the commonly used transformation methods, such as *Agrobacterium*-mediated transformation or biolistics (particle bombardment). When working with inducible promoters (see below), getting rid of the editing machinery after mutagenesis of the target region is not necessary. However, in other cases, it is strongly recommended to remove the cassette as soon as possible. It allows minimization of the number of off-target mutations and also possible interference of *Sp*Cas9 with transcription in cases when *Sp*Cas9 stays stably bound in an off-target site with the sgRNA complementarity insufficient for cleavage.
When working with generative reproducing models, the cassette can be outcrossed in the next generations. Alternatively, it is possible to remove the cassette by targeted excision. Such self-excision systems are based on either CRE/lox or other site-specific recombinases, or even on Cas9 itself when it is provided with additional sgRNA targeting the borders of the transgenic cassette (Sheva *et al.*, 2020^P^).
(ii) To avoid the need for cassette removal, a transient expression system might be considered which is usually based on some viral vector or DNA/RNA introduced via particle bombardment. These vectors do not tend to integrate into the plant genome and, as extrachromosomal elements, they can be eliminated from the cell progeny (Y. Zhang *et al.*, 2016^P^; L. Chen *et al.*, 2018^P^).
(iii) Alternatively, mRNAs encoding for *Sp*Cas9 and sgRNA or even *in vitro* assembled RNPs consisting of the *Sp*Cas9 with sgRNA might be delivered. Delivering as RNPs can also minimize the number of off-targets (Woo *et al.*, 2015^P^; Malnoy *et al.*, 2016^P^; Subburaj *et al.*, 2016^P^).
(iv) For some purposes, it is possible to graft wild-type shoots to transgenic donor rootstock, where, for example, Cas9 and sgRNA transcripts are fused to a tRNA-like sequence motif that moves RNAs from the donor transgenic rootstock to the grafted wild-type shoot (Yang *et al.*, 2023^P^).

### What vector design to choose

Optimization of the system can be achieved at many levels for individual needs, for example by choosing the promoter and terminator variants, a codon-optimized variant of *Sp*Cas9, the presence or absence of nuclear localization signal (NLS), etc. Some of those we discuss below.

#### *Promoter for* Sp*Cas9*

The choice of a suitable promoter mostly depends on the model/transformed cell type. A constitutive promoter, such as cauliflower mosaic virus P35S or a ubiquitin promoter, can be used for somatic differentiated cells, cell lines, or tissue cultures. Various plant tissue-specific promoters have been used for specific *Sp*Cas9 applications (Singha *et al.*, 2021^P^; Rahman *et al.*, 2022^P^). Inducible promoters, driven by chemical agents (e.g. steroids, tetracycline, insecticide, or ethanol) or optogenetic tools can be used to avoid excessive Cas9 activity (Zuo and Chua, 2000^P^; Ochoa-Fernandez *et al.*, 2020^P^; Omelina *et al.*, 2022^P^). For floral dip transformation of *A. thaliana*, the egg cell-specific promoter is the optimal choice which may produce homozygous mutants for multiple target genes within a single generation (Wang *et al.*, 2015^P^), although it is usually not so fast. In addition to ‘traditionally’ used promoters, synthetic promoters might also be used, as they offer the potential to overcome some of the limitations of native promoters (Ali and Kim, 2019^P^; Yasmeen *et al.*, 2023^P^).

#### *Type of* Sp*Cas9 gene*

The most widely used *Sp*Cas9 is a codon-optimized version for *A. thaliana* (Fauser *et al.*, 2014^P^), but many other variants also exist, for example one containing 13 introns in the *Sp*Cas9 coding sequence for more stable expression (Grützner *et al.*, 2021^P^). To overcome the limitation of the PAM 5'-NGG-3' sequence in the selection of target sites (e.g. when editing A–T-rich promoters and terminators), mutated *Sp*Cas9 variants were prepared to recognize different PAM sequences. For example, *Sp*Cas9-NG and xCas9 were demonstrated to recognize 5'-NG-3' PAM sequences in human cell lines (Hu *et al.*, 2018^O^; Nishimasu *et al.*, 2018^O^). Also, some other features of *Sp*Cas9 have been altered, such as the addition or reduction of NLSs (Osakabe *et al.*, 2016^P^).

Moreover, from the same type II CRISPR system, there is also a frequently used *Sa*Cas9 variant from *Staphylococcus aureus*, and from the type V system, *Lb*Cas12a, also known as *Lb*Cpf1 from *Lachnospiraceae bacterium* ND2006 (Huang and Puchta, 2021^P^).  A recent study also identified 188 new CRISPR-linked gene modules (Altae-Tran *et al.*, 2023^O^), opening up more possibilities without the need for engineering new variants from scratch. Nevertheless, those and other variants differ not just by recognizing different PAM sequences but also by different interactions with the target sequence, the cut position, or the dissociation rate from the cleavage products. For more information, we recommend reading the recent plant review by Huang and Puchta (2021^P^) and Altae-Tran *et al.* (2023O).

#### Number and arrangement of sgRNA genes

The CRISPR/Cas9 system is often used to target many targets at once. To do this, more than one sgRNA needs to be introduced into the cell. One of the possible methods is to clone individual sgRNAs with their promoters to a final vector (Hu *et al.*, 2019). However, this approach has the disadvantage of rapidly increasing the size of the resulting vector, even though using prepared vectors for fast cloning speeds up the process (Hu *et al.*, 2019^P^). On the other hand, it allows the expression of individual sgRNAs using tissue-specific promoters. Another more convenient method is to organize multiple sgRNAs to a polycistron transcribed from one Pol III promoter. Such organization saves space in the vector as individual sgRNAs are released by processing of the transcript by endogenous RNases P and Z that cleave out tRNAs located in between (Dong *et al.*, 2017^O^). Moreover, when combined with the Golden Gate (Engler *et al.*, 2008^O^; Hu *et al.*, 2019^P^; Kim *et al.*, 2021^P^) or GoldenBraid (Sarrion-Perdigones *et al.*, 2011^P^; Vazquez‑Vilar *et al.*, 2016^P^) cloning system, it is quick to change guide regions in the individual vectors (Vazquez‑Vilar *et al.*, 2016^P^; Hu *et al.*, 2019^P^; Kim *et al.*, 2021^P^).

## Other uses of the *Sp*Cas9 system

Besides using *Sp*Cas9 for targeted cutting followed by random or pre-designed repair, fully inactivated *Sp*Cas9 can also be used for other purposes.

One of the first engineered variants of *Sp*Cas9 was a catalytically inactive mutant, dCas9. It can still target the DNA in the same way as native *Sp*Cas9, but it is unable to cleave (Qi *et al.*, 2013^O^). This makes dCas9 a versatile tool. When fused with fluorescent molecules, it can be used for imaging of specific genomic loci in living organisms (Anton *et al.*, 2016^O^; Ye *et al.*, 2017^O^; Fujimoto and Matsunaga, 2017^P^; B. Chen *et al.*, 2018^O^; Khosravi *et al.*, 2020^P^; Clow *et al.*, 2022^O^; van Staalduinen *et al.*, 2023^O^). Alternatively it can be used to modulate gene expression, either directly by fusing to activators or repressors of transcription or by attracting them by aptamers incorporated within the sgRNA. Transcription in the target region can also be influenced indirectly by fusing dCas9 with various chromatin-modifying enzymes (Dominguez *et al.*, 2016^O^;  Adli, 2018^O^; Xu and Qi, 2019^O^; Karlson *et al.*, 2021^P^). In all these applications, special care must be taken for off-targets because the presence of a PAM and a complementary stretch of only 9 nt upstream from a PAM are sufficient for strong binding (Sternberg *et al.*, 2014^O^; Boyle *et al.*, 2017^O^).

## Conclusion

Since its discovery, the CRISPR/Cas9 system quickly became a widely used tool for both basic research and breeding new crop varieties for agriculture. This is mostly thanks to the possibility of gene editing directly in the genome with a fast and straightforward preparation of the sgRNA for (relatively) precise targeting. Although many researchers use it on a daily basis, there are still many people who are afraid to start working with it. A huge amount of data is available on the CRISPR/Cas9 action mechanism, but not all important aspects have been fully adopted. Novices and experienced researchers alike rely on many software programs available for searching on-targets and predicting off-targets for selecting sgRNA. Unfortunately, these predictions are often misleading, and the computational results lack the predictive power for plant models for several reasons. The greatest drawback is that the prediction tools were trained mostly on animal cell lines. The tools provide accurate predictions for the lines used for training, but, unfortunately, as far as we are aware, no such tool has been trained and optimized for plant models. Even though the CRISPR/Cas9 actions are principally the same in any cell, the type of cleavage at the target (blunt/staggered) and the RuvC post-cleavage processivity are likely to be influenced by chromatin structure and dynamics, which may be cell type and species specific. Also, above all, the availability of individual DNA repair pathways affects the mutagenesis output the most. It depends not only on the respective organism but also on specific experimental conditions and, importantly, on whether the editing occurs in proliferating or differentiated cells.

There are still many gaps in our knowledge. We still do not fully understand many steps of the whole editing process, especially regarding the repair of CRISPR/Cas9-induced DNA breaks. For example, as suggested by the dramatic differences in prime editing between monocots and dicots, the repair pathways involved in nick repair might differ between those two clades. Understanding individual factors and pathways will lead to the possibility of developing more efficient CRISPR/Cas9 systems for plant breeding and basic research. Future plant-oriented research is needed, as only a limited amount of information can be transferred from animal/yeast models.
